# Stronger expression of crassulacean acid metabolism (CAM) requires effective cuticular transpiration barriers but not necessarily strong succulence

**DOI:** 10.1111/nph.70909

**Published:** 2026-01-23

**Authors:** Thibaud F. E. Messerschmid, Jurriaan M. de Vos, Susanne E. Hamburger, Jessica A. Berasategui, Gudrun Kadereit

**Affiliations:** ^1^ Botanischer Garten München‐Nymphenburg Staatliche Naturwissenschaftliche Sammlungen Bayerns Menzinger Str. 65 80638 München Germany; ^2^ Department of Environmental Sciences ‐ Botany University of Basel Schönbeinstr. 6 4056 Basel Switzerland; ^3^ Institut für Molekulare Physiologie Johannes Gutenberg‐Universität Mainz Anselm‐Franz‐von‐Bentzel‐Weg 9a 55099 Mainz Germany; ^4^ Prinzessin Therese von Bayern‐Lehrstuhl für Systematik, Biodiversität & Evolution der Pflanzen Ludwig‐Maximilians‐Universität München Menzinger Str. 67 München 80638 Germany

**Keywords:** *Aeonium*, Canary Islands, Crassulaceae, crassulacean acid metabolism, cuticle, functional traits, minimum conductance, succulence

## Abstract

- Discovering functional and evolutionary interdependencies of hydraulic traits and crassulacean acid metabolism (CAM) is crucial to understand CAM phenotype diversity and convergence. In complex traits such as CAM, the co‐option of associated traits strongly impacts the evolutionary outcome. Here we study *Aeonium* (Crassulaceae), a diverse Macaronesian genus that exhibits a broad array of CAM expression, focusing on two CAM‐associated traits, minimum conductance (*g*_min_) and succulence.
- At the heart of the study, there are two experiments: a comparative cultivation experiment to monitor nocturnal acidification (ΔH^+^) under drought and heat treatments and a leaf‐drying curve experiment to quantify *g*_min_. Our study group was comprehensively sampled to cover its phylogenetic and ecological diversity.
- We found a consistently negative correlation of *g*_min_ and ΔH^+^, indicating a critical role for the cuticle in the function of CAM. Although ΔH^+^ and succulence were overall not positively correlated, we found evidence that more succulent species remain in the CAM mode when stress is relaxed.
- We conclude that there is a tight evolutionary link between cuticular transpiration barrier properties and CAM performance. Thereby, the stronger CAM plants express diurnal stomatal closure typical of CAM, the more they may optimise water‐use efficiency through reduced *g*_min_.

## Introduction

The survival of a plant strongly depends on the interplay between its traits and the environment. Any survival strategy is the result of the evolution of an entire suite of traits ancestral to the respective lineage. In particular, traits as complex as crassulacean acid metabolism (CAM) that presumably evolved over a long time period are associated with other traits (Niechayev *et al*., 2019) that collectively determine the fitness of a plant with CAM. More specifically, CAM – that is, the nocturnal assimilation of CO_2_ into four‐carbon organic acids which serve as carbon storage for daytime photosynthesis – has been known to be associated with succulence (Osmond, 1978; Griffiths & Males, 2017), most evidently because larger vacuoles potentially provide a higher storage capacity for organic acids (Nuernbergk, 1961; Edwards, 2019). Similar to CAM, succulence itself is an effective adaptation to environmental water scarcity, particularly seasonal drought (Eggli & Nyffeler, 2009; Males, 2017).

The general adaptive value of CAM results from the opening of stomata during the cooler and more humid night hours (extending into dawn) instead of during the day (Lüttge, 1987). But plants with CAM usually take up a proportion of CO_2_ during the day via the more common C_3_ photosynthetic pathway, not exclusively during the night. There is therefore a continuum in the expression of CAM between C_3_ photosynthesis with possible induction of CAM by stressful conditions (i.e. facultative CAM) and constitutive CAM which itself is usually also associated with a variable proportion of daytime C_3_ photosynthesis (Winter, 2019). The extent to which CAM plants are able to avoid high daytime transpiration and to remain isohydric depends on the ability to minimise water loss through the cuticle and through ‘leaky stomata’ (Kerstiens, 1996; Burghardt & Riederer, 2006; Machado *et al*., 2021; Burlett *et al*., 2025). Therefore, selection towards minimising the cuticular conductance and minimal residual stomatal conductance after full closure might be strong in the context of CAM evolution. Cuticular conductance is usually constant, but has been shown to vary with temperature (e.g. Bueno *et al*., 2019), turgor (Boyer, 2015), soil water availability, and leaf age (both Duursma *et al*., 2019). In contrast to cuticular conductance, stomatal conductance is variable and regulated by plants through stomatal opening and closure. In leaves that lack astomatous surfaces entirely, minimum conductance (commonly abbreviated as *g*_min_) is usually measured by inferring leaf‐drying curves from detached leaves. It can serve as a ‘potentially strong determinant of drought tolerance’ (Sack & Scoffoni, 2010), although it is only a proxy for real minimum conductance, that is, the conductance to water measured in an intact leaf attached to a plant after full closure of stomata (Duursma *et al*., 2019; Márquez *et al*., 2022). This real *g*_min_ is arguably more relevant than cuticular conductance alone because a plant's ability to close stomata directly influences the actual rate at which water is lost by a drying organ (Burghardt & Riederer, 2003) and may therefore, together with cuticular transpiration barrier properties, determine the survival of a plant (Burlett *et al*., 2025).

Past studies have highlighted various characteristics that may minimise transpiration as hallmark features of plants with CAM (e.g. low surface area: volume ratio and low stomatal density (Winter *et al*., 2015) or a thick cuticle (Niechayev *et al*., 2019)). Indeed, Barrera Zambrano *et al*. (2014) found a negative relationship between the proportion of CO_2_ taken up at night (as a measure of CAM activity) and stomatal density as well as maximum stomatal conductance in *Clusia*, highlighting the relevance of stomatal properties for CAM function. However, only little is known about the role of cuticular properties in CAM physiology, and to our knowledge *g*_min_ has never been investigated in the context of CAM photosynthesis. The ecophysiological relevance of *g*_min_ for the overall water loss of a photosynthetically active organ with CAM may be strikingly variable, depending on whether CAM is expressed in a facultative or obligate manner and depending on the proportion of CO_2_ being fixed directly by the C_3_ pathway. While weak CAM or C_3_‐CAM plants might never experience prolonged periods of daytime stomatal closure, constitutive CAM plants or plants in a CAM‐idling mode (i.e. stomatal closure day and night with re‐fixation of respiratory CO_2_) keep stomata closed at least during hot and dry atmospheric conditions. The latter should therefore be much more dependent on effective cuticular transpiration barrier properties for their water relations than plants with carbon gain predominantly from the C_3_ pathway. We therefore hypothesise that *g*_min_ should be negatively correlated with the extent to which CAM is expressed.

Succulence undoubtedly plays an important role in the water relations of plants with CAM, too, because it relates to the amount of utilisable water, that is, the amount of water that may be mobilised and used for photosynthesis and nutrient transport during periods without water supply (von Willert *et al*., 1992). As mentioned above, succulence is the functional basis of an efficient CAM pathway and maximises the nocturnal storage capacity for the organic acids that serve as a carbon source for daytime photosynthesis. We therefore hypothesise a positive correlation between succulence and the extent to which CAM is expressed.

*Aeonium* (Crassulaceae) is a morphologically and ecologically highly diverse genus of 41 species that largely radiated on the Canary Island archipelago (Jorgensen & Olesen, 2001; Messerschmid *et al*., 2023). This group with well‐established monophyly (Mes, 1995; Mort *et al*., 2002) is appropriate to test the two hypotheses that we propose here because it exhibits enormous variability of succulence (Supporting Information Fig. S1) and CAM activity (Lösch, 1990; Pilon‐Smits *et al*., 1992; Mort *et al*., 2007). Especially the work of Rainer Lösch (1984, 1990) on CAM in *Aeonium* revealed a high plasticity of the proportion of day‐ and night‐time CO_2_ assimilation within species, depending on environmental factors, especially temperature. This plasticity of CAM expression in response to environmental conditions is usually referred to as facultative CAM if the factor triggering CAM expression is water‐deficit stress (Winter, 2019). In the context of CAM, temperature is commonly seen as an environmental factor modulating the influence of other factors (Lüttge, 2004). For example, higher temperatures are usually associated with increases in the leaf‐atmosphere vapour pressure deficit (Medina, 1982), that is, the driving force of transpiration. Heat may thereby act as a signal for CAM induction in order to increase water‐use efficiency, especially in light of the fact that CAM species are usually highly sensitive to increases in vapour pressure deficit (Lange & Medina, 1979; Males & Griffiths, 2017).

In this study, we aim to increase the knowledge of the cuticular biology of plants with CAM, which remains largely unknown compared to the stomatal biology of CAM plants. To our knowledge, *g*_min_ has not been tested for any representative of Crassulaceae after the very first leaf‐drying curves were published for, among others, *Hylotelephium telephium* subsp. *maximum* (Pisek & Berger, 1938). It seems to be overall understudied for succulent plants (Schuster *et al*., 2017). We here investigate correlations between succulence, *g*_min_, and CAM activity by means of leaf‐drying experiments and extensive climate chamber experiments involving temperature and drought treatments. Specifically, we test whether *g*_min_ is negatively correlated and whether succulence is positively correlated with CAM activity as quantified by the titratable acidity of *Aeonium* leaf samples collected under well‐defined environmental conditions. A recent genus‐wide phylogeny (Messerschmid *et al*., 2023) and comprehensive data from field observations of the spatial distribution of *Aeonium* species (dos Santos *et al*., 2024) furthermore allow us to analyse the results of our physiological measurements in a phylogenetic and ecological framework.

## Materials and Methods

### Taxon sampling and plant material

As many species of *Aeonium* as possible were included in our climate chamber and leaf‐drying experiments. The final taxon sample covered a broad range of growth forms as described by Liu (1989) and dos Santos *et al*. (2022), photosynthetic diversity (Lösch, 1990), and sections of the genus. The taxonomy of *Aeonium* followed Bañares Baudet (2015) or Liu (1989) for species not covered by the former. Of the 41 species and eight additional intraspecific taxa of *Aeonium*, 32 species and four subspecies were sampled here for their CAM physiology (see ‘Climate chamber experiment’ below) as well as succulence and *g*_min_ (see ‘Leaf‐drying curves’ below). The plant material used is summarised in Table S1 along with the growth form of each species. Due to differences in growth patterns and growth rates among *Aeonium* species, not all species could be propagated in equally high numbers (see Table S1) for the climate chamber experiment outlined in the following section.

### Climate chamber experiment

**Fig. 1:**
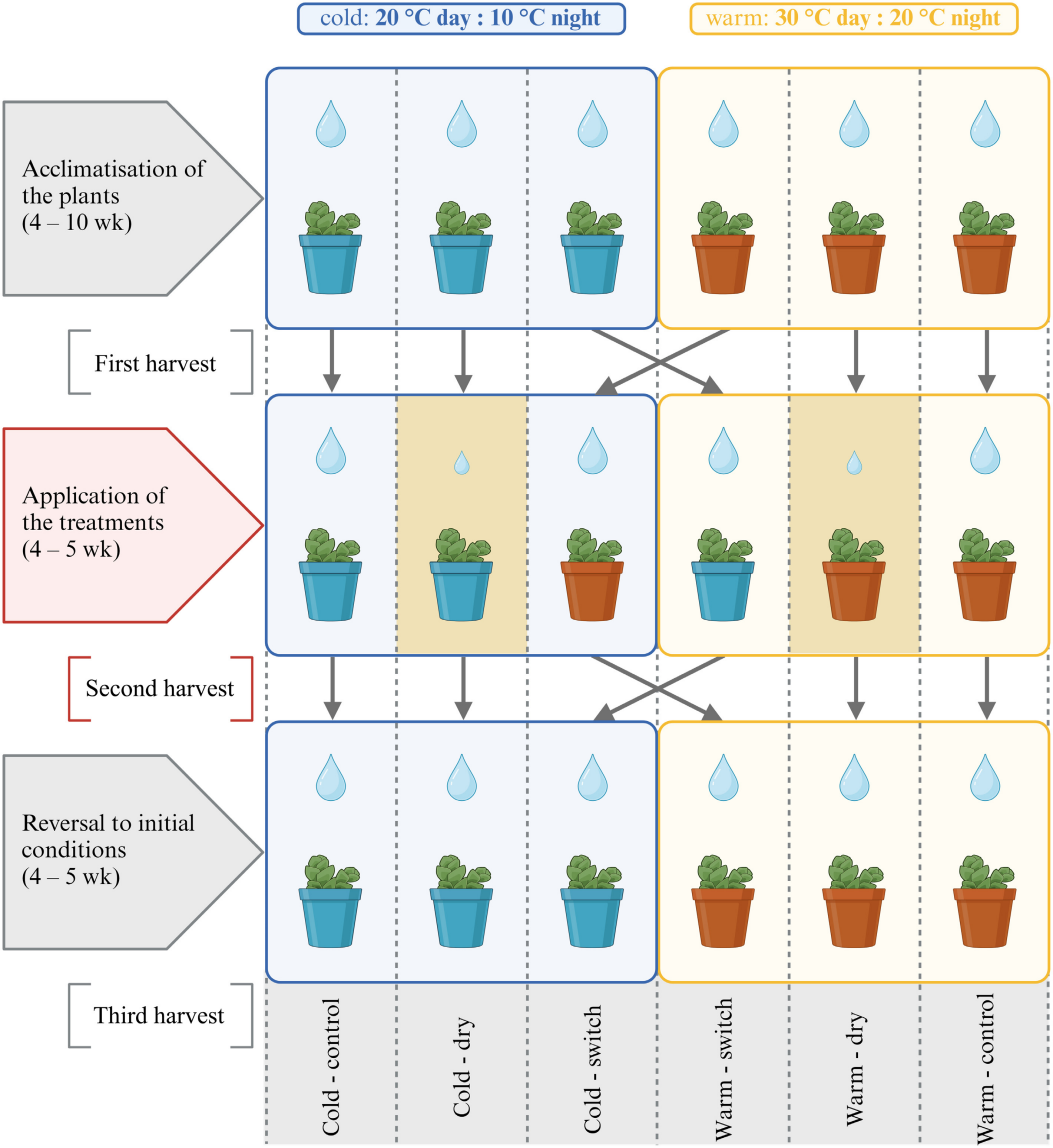
Outline of the climate chamber experiment conducted with all sampled species of *Aeonium* in chronological order from top to bottom. Leaves were harvested for analysis of titratable acidity in the evening and the subsequent morning after each of the three experimental phases. The treatment phase and corresponding second harvest are highlighted in red. This figure was created in BioRender (BioRender.com/z5i04v6).

Individual plants were propagated as vegetative cuttings or, if not possible (*A. glandulosum* (Aiton) Webb & Berthel. and *A. tabuliforme* Webb & Berthel.), from seeds. Those nine taxa with only one tested individual (see number of replicates in Table S1) were unsuitable for propagation at the beginning of the study and underwent the climate chamber experiment as a single potted plant. Propagated individual plants were potted in square 8 × 8 cm plastic pots in cultivation substrate and kept under favourable conditions for at least 2 wk to ensure proper rooting, viability and growth before the beginning of the experiment. In case of sufficiently high numbers of replicates per taxon, the individuals were subdivided into six different experimental groups (columns in Fig. 1): cold – control, cold – dry, cold – switch, warm – control, warm – dry, and warm – switch. The nine taxa with only one available replicate were all assigned to the cold – switch group, so that cold – switch was the one group that all taxa underwent. The reason for this was that the drought treatment of the warm – dry group was too severe for some of the plants and that the CAM induction was stronger after the heat treatment (cold – switch) than after the drought treatment (cold – dry) in the first two taxa that we tested simultaneously (i.e. *A. goochiae* Webb & Berthel. and *A. haworthii* Webb & Berthel.). In the first phase of the experiment (top line in Fig. 1), plants were acclimatised for four up to 10 wk to a temperature regime of 20°C : 10°C day/night (groups cold – control, cold – dry and cold – switch) or 30°C : 20°C day : night (groups warm – control, warm – dry and warm – switch), respectively. All plants were watered regularly, usually three times a week, depending on individual demands. The duration of day and night followed a 12‐h photoperiod throughout the entire experiment, with light intensities between 85 and 360 μmol m^−2^ s^−1^ at the level of leaf rosettes. After initial acclimatisation, leaf samples were collected from each plant within the last 90 min of the light period and within the last 90 min of the subsequent dark period (first harvest in Fig. 1) to quantify nocturnal acidification (ΔH^+^) in the context of CAM activity (see subsequently). Leaf samples were immediately snap frozen in liquid nitrogen and stored at −20°C until acidity titration.

After the first harvest, plants (except for the controls) were subjected to a treatment according to their experimental groups. Plants of the groups cold – dry and warm – dry were only watered once a week (drought treatment) while cold – switch and warm – switch plants were displaced from the cold to the warm climate chamber or vice versa (temperature treatment). This treatment phase lasted 4–5 wk and was terminated with another harvest of leaf samples as described above, taking care that the plants with drought treatment had not received water for an entire week. Plants were then placed back to experience the same conditions as in the initial acclimatisation phase of the experiment for another 4–5 wk. After this reversal phase, a third harvest of leaf samples for acidity titration was conducted. Consequently, six leaf samples were harvested for each individual plant throughout the course of the climate chamber experiment: one evening and one morning sample at the end of each of the three phases, that is acclimatisation, treatment and reversal. The plants typically continued to grow throughout the course of the climate chamber experiment so that leaves could be sampled for titratable acidity measurements that were developed during the time that a treatment or reversal of a treatment was applied.

### Titration of leaf acidity

For the determination of the flux in titratable acidity in the leaf tissues to quantify CAM activity, the frozen samples were finely chopped into pieces of ≤ 1 mm edge length, allowing for the sample to thaw in the process. Subsequently, 50 mg (±20 mg) of each sample was weighed into a test glass tube and submerged in 3.5 ml of 20% ethanol. The test glasses were incubated in a water bath at 65°C for 1 h before being thoroughly vortexed and cooled to room temperature. Each sample was then split up into three smaller test glasses serving as technical replicates, containing 1 ml of the alcoholic extract without any leaf pieces, and 50 μl of bromothymol blue was added as pH indicator to each replicate. Titration was performed by stepwise addition of 10 mM NaOH and thorough vortexing until the colour of the indicator signalled neutrality of the solution. Acid accumulation overnight (ΔH^+^) was calculated as the absolute difference of free acid concentration per gram leaf fresh weight for each pair of evening and morning samples.

### Leaf‐drying curves

For the inference of drying curves, young but fully expanded leaves were collected in the glasshouse from the mother plants of the individuals propagated for the climate chamber experiment or from the experimental plants themselves before their involvement in the climate chamber experiment, for those taxa with only one available plant (see number of replicates in Table S1). Six leaves per taxon were collected as biological replicates. Only for *A. canariense* subsp. *christii* (Burchard) Bañares, *A. davidbramwellii* H.Y.Liu, *A. gorgoneum* J.A.Schmidt, *A. leucoblepharum* Webb ex A.Rich., *A. percarneum* (Murray) Pit. & Proust and *A. undulatum* Webb & Berthel., up to 12 leaves were collected because the climate chamber experiment was repeated for these taxa at a later time point to increase the sample size. Drying curves of detached and fully rehydrated leaves were obtained as described by Burghardt & Riederer (2003) using molten paraffin wax (Richard‐Allan Scientific, Kalamazoo, MI, USA) to seal the cut leaf bases after overnight rehydration. Leaves were scanned to determine leaf area at water saturation, and the double projected leaf area was used to calculate transpiration rates and conductances, considering that both leaf sides carry stomata in *Aeonium* (Liu, 1989). Minimum conductance (*g*_min_, mmol m^−2^ s^−1^) was calculated as the mean of all conductance values measured beyond the turgor loss point and before any lethal dehydration which is typically reached at a relative water deficit (RWD) of *c*. 0.7. After recording several values of *g*_min_ beyond the turgor loss point, the weight of the paraffin wax seal was noted to correct for leaf mass, and leaves were dried completely overnight in an oven between 70 and 80°C to determine their dry weight. The degree of succulence (DS; g m^−2^) was calculated following Delf (1912) as the quotient of water mass in a fully hydrated leaf and leaf surface area (i.e. the double projected leaf area at full hydration). In addition, leaf thickness (LT; mm) was measured with a digital caliper on fresh leaves of each mother plant in the greenhouse, taking care that these had been watered on the previous day.

For *A. arboreum* subsp. *holochrysum* var. *rubrolineatum* (Svent.) H.Y.Liu, *A. aureum* (C.Sm. ex Hornem.) T.Mes, *A. glandulosum*, *A. lindleyi* Webb & Berthel. subsp. *lindleyi* and *A. volkeri* E.Hern. & Bañares (see Fig. S1), leaf shrinkage curves were inferred in addition to the leaf‐drying curves in order to correct for leaf area loss upon drying in the calculation of transpiration rates and conductances. For leaf shrinkage curves, detached leaves were laid between several sheets of blotting paper to avoid curling of the leaves while drying. Weight and leaf area were measured in continuously increasing time intervals and in the end after overnight incubation in a drying oven at 90°C between two fire‐proof glass plates to determine weight and area at complete desiccation. Finally, all leaf‐drying curves were corrected by accounting for percentage leaf area loss as a function of RWD at each measurement. Species that were not sampled for leaf shrinkage curves were assigned to one of the species with a known leaf shrinkage curve (see Table S1) on the basis of similar leaf shape in order to carry out leaf‐drying curve corrections for these species, too.

Our measurements of *g*_min_ and DS on the one hand and ΔH^+^ on the other hand were independent of each other, because for drying curves, leaves were collected in the glasshouse, and for acid titration, leaves were collected in the climate chambers. Therefore, simple linear correlation analyses were performed with the means of these variables on the taxon level.

### Phylogenetic analysis of trait evolution

Phylogenetic analysis was based on the maximum clade credibility *Aeonium* species tree from Messerschmid *et al*. (2023), which had been inferred from long‐range ddRAD loci and was well supported. The phylogeny was pruned to only contain the taxa that were sampled for the respective climate chamber groups and phases. In this process, data for both subspecies of *A. lindleyi* (see Table S1) were lumped together because *A. lindleyi* subsp. *viscatum* (Bolle) Bañares had not been sampled for the phylogeny (Messerschmid *et al*., 2023). All phylogenetic analyses were carried out using the packages ape (Paradis & Schliep, 2019), geiger (Pennell *et al*., 2014), nlme (Pinheiro *et al*., 2024), and phytools v.2.1‐1 (Revell, 2024) in R. Using the *contMap* function in *phytools*, the evolution of ΔH^+^ as a quantification of CAM expression for each group and phase of the climate chamber experiment was mapped onto the phylogeny. To test for phylogenetic signal, Pagel's λ was calculated for *g*_min_, DS, LT, and ΔH^+^ values from each experimental phase using the *phylosig* function in *phytools*. Here, λ = 1 indicates that the phylogenetic pattern is well‐approximated by an evolutionary model of Brownian motion (BM, that is, species covary in linear proportion to their shared branch length in the phylogeny). Conversely, λ = 0 indicates no phylogenetic signal (i.e. differences among species arise along the terminal branches of the phylogeny, and the phylogeny does not inform covariance among species). The λ values were tested for significant differences of λ > 0 using the likelihood‐ratio test implemented in *phylosig* using default settings.

Finally, climatic data for each occurrence point on the Canary Islands were taken from dos Santos *et al*. (2022), who had based it on CHELSA v.1.2 (Karger *et al*., 2017) for localities mostly surveyed newly in the field (10 180 observations). We then used phylogenetic generalised least squares (PGLS) regression analysis of the ΔH^+^ and *g*_min_ data against all available bioclimatic variables and potential seasonal solar radiation. Again, the phylogeny was pruned to only contain those taxa with available occurrence and ΔH^+^/*g*_min_ data. For this analysis, ΔH^+^ data were restricted to the values retrieved from plants that had received a heat treatment, that is, the treatment phase of group cold – switch, because all taxa underwent this treatment and ΔH^+^ was most variable across taxa in this data subset. In addition, PGLS regression analyses were also performed for *g*_min_ and DS against ΔH^+^ data for all phases of the climate chamber experiment.

## Results

### Titratable acidity and leaf‐drying curves

**Fig. 2:**
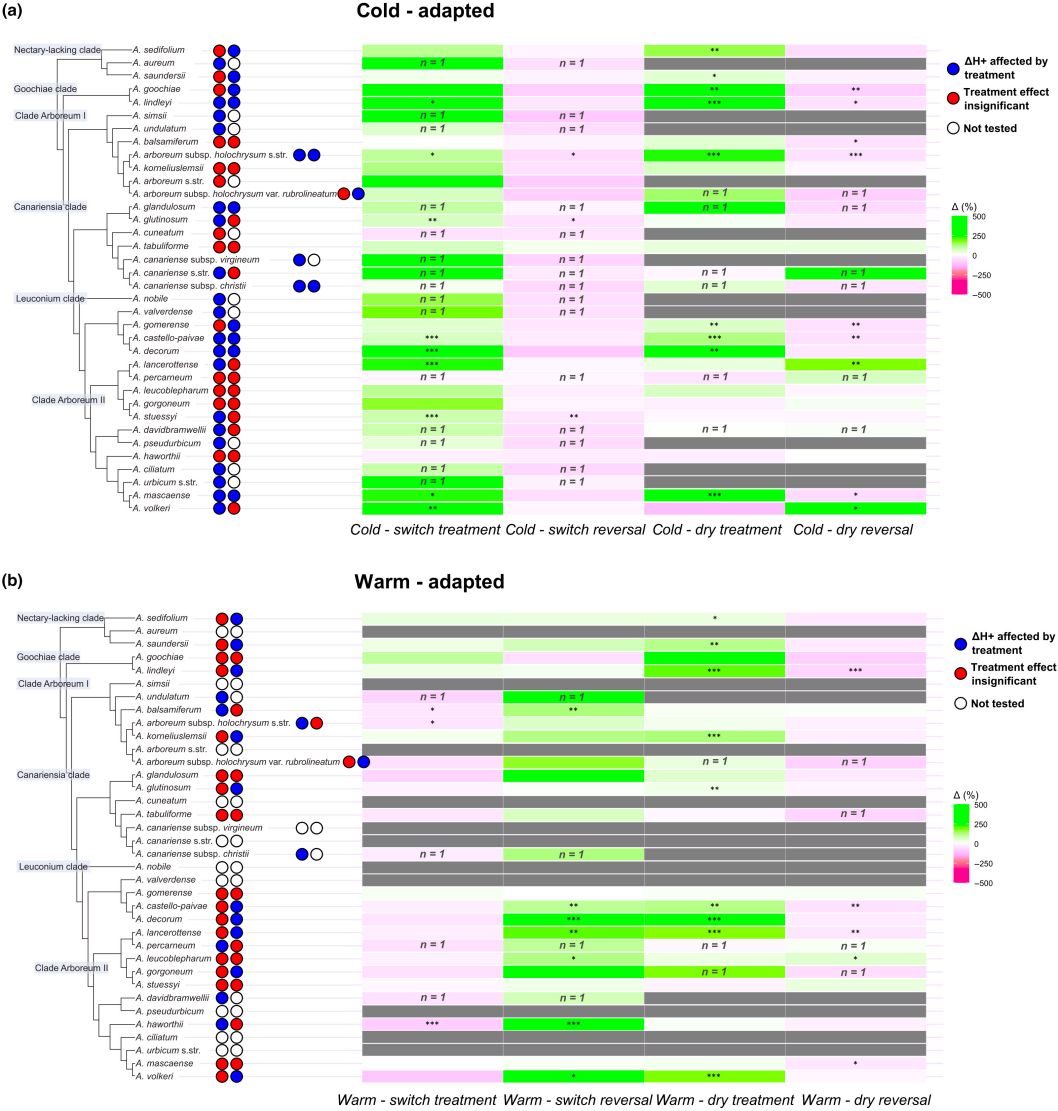
Heatmap of nocturnal acid accumulation (ΔH^+^) in the *Aeonium* climate chamber experiment for (a) cold‐acclimatised plants and (b) warm‐acclimatised plants. The heatmap shows percentage increases (green) or decreases (magenta) of ΔH^+^ relative to the control (for treatments) and relative to the treatments (for reversals) for each taxon. Columns represent experimental phases (see bottom for description). Each taxon is colour‐coded with two dots representing the effect of the temperature (left dot) or drought (right dot) treatments or their respective reversals on ΔH^+^. Effects are classified as significant (or obvious in taxa with *n* = 1; blue) or non‐significant (red) on the basis of two‐sided *t*‐tests. Missing data is shown by white dots and grey cells.

Altogether 515 plants from 36 *Aeonium* taxa underwent the climate chamber experiment, of which 490 plants were fully sampled for acid titration throughout the experiment. The remaining 25 plants either died towards the end of the experiment, or corresponding samples were lost. Overall, 1520 ΔH^+^ (μmol g^−1^) data points were obtained from the acid titration data. Values for ΔH^+^ (summarised in Table S2 with raw data of titratable acidity) ranged from −56.95 μmol g^−1^ (i.e. nocturnal reduction of acidity; after warm – switch treatment in *A. volkeri*) to 224.88 μmol g^−1^ (i.e. nocturnal acidification; after cold – dry treatment in *A. arboreum* subsp. *holochrysum*). None of the taxa had consistently negative ΔH^+^ values, but two taxa only had ΔH^+^ values < 15 μmol g^−1^, that is *A. aureum* and *A. canariense* subsp. *virgineum*. However, we did not consider them C_3_ species because they were only represented by one plant, and individuals of other taxa with CAM showed similarly low ΔH^+^ ranges. Most taxa could be characterised as low‐level constitutive CAM with facultative upregulation of CAM expression. Thus, most taxa showed a significant increase in CAM activity in the heat and/or drought treatments (including taxa with only one replicate, blue dots in Fig. 2). The overall more succulent Leuconium clade + clade Arboreum II had fewer significant decreases of ΔH^+^ upon reversal of heat or drought stress after cold acclimatisation than the remaining *Aeonium* species (Fig. 2a), some of them even enhancing CAM activity under the relaxed experimental conditions. Only three taxa (i.e. *A. arboreum* s.str., *A. cuneatum*, and *A. tabuliforme*) did not react significantly to any imposed stress.

Drying curves were carried out on 247 individual leaves from the same taxa and accessions as for the climate chamber experiment. Medians of *g*_min_, DS, and LT are given in Table 1 (all raw data in Table S3). Individual values of *g*_min_ diverged by two orders of magnitude, between 0.07 mmol m^−2^ s^−1^ (*A. glutinosum*) and 5.21 mmol m^−2^ s^−1^ (*A. sedifolium*), and DS ranged from 257.9 g m^−2^ (*A. goochiae*) to 2272.7 g m^−2^ (*A. nobile*). LT was significantly correlated with DS (Fig. S2) and ranged from 0.85 mm (*A. goochiae*) to 7.24 mm (*A. nobile*).

**Table 1:** Medians and interquartile ranges of minimum conductance (*g*_min_), degree of succulence (DS), and leaf thickness (LT) for each investigated species.

Species	*g*_min_ (mmol m^−2^ s^−1^)	DS (g m^−2^)	LT (mm)
*Aeonium arboreum* subsp. *arboreum*	0.40 (±0.04)	657.1 (±99.6)	1.67 (±0.18)
*A. arboreum* subsp. *holochrysum* var. *holochrysum*	0.38 (±0.11)	566.4 (±35.2)	1.18 (±0.06)
*A. arboreum* subsp. *holochrysum* var. *rubrolineatum*	0.13 (±0.04)	507.2 (±39.8)	na
*A. aureum*	0.60 (±0.20)	669.2 (±83.2)	1.02 (±0.23)
*A. balsamiferum*	0.69 (±1.26)	618.4 (±26.4)	1.78 (±0.06)
*A. canariense* subsp. *canariense*	1.04 (±0.89)	1012.0 (±68.0)	na
*A. canariense* subsp. *christii*	0.48 (±0.60)	1263.1 (±477.2)	5.43 (±0.14)
*A. canariense* subsp. *virgineum*	1.03 (±0.48)	1220.6 (±94.4)	3.86 (±0.17)
*A. castello‐paivae*	0.10 (±0.01)	570.7 (±61.8)	3.63 (±0.14)
*A. ciliatum*	0.66 (±0.12)	971.1 (±30.3)	2.87 (±0.10)
*A. cuneatum*	0.43 (±0.08)	620.7 (±28.8)	2.82 (±0.23)
*A. davidbramwellii*	0.53 (±0.60)	1330.2 (±607.5)	3.98 (±0.21)
*A. decorum*	2.15 (±0.56)	903.3 (±84.6)	3.79 (±0.23)
*A. glandulosum*	0.37 (±0.14)	535.6 (±62.2)	3.13 (±0.12)
*A. glutinosum*	0.07 (±0.00)	766.6 (±99.6)	3.40 (±0.27)
*A. gomerense*	0.10 (±0.01)	712.9 (±49.3)	3.07 (±0.12)
*A. goochiae*	0.76 (±0.40)	349.7 (±96.6)	1.22 (±0.26)
*A. gorgoneum*	0.60 (±0.20)	1185.8 (±394.4)	3.18 (±0.09)
*A. haworthii*	0.10 (±0.03)	937.4 (±30.4)	3.52 (±0.10)
*A. korneliuslemsii*	0.24 (±0.05)	901.8 (±59.0)	2.15 (±0.08)
*A. lancerottense*	0.14 (±0.04)	699.7 (±48.5)	3.37 (±0.15)
*A. leucoblepharum*	2.27 (±0.96)	949.7 (±930.4)	2.86 (±0.15)
*A. lindleyi*	0.69 (±3.34)	939.6 (±130.1)	3.50 (±0.77)
*A. mascaense*	0.66 (±0.23)	1047.4 (±165.2)	3.20 (±0.23)
*A. nobile*	0.24 (±0.01)	1983.8 (±154.6)	6.69 (±0.29)
*A. percarneum*	0.42 (±0.46)	1095.9 (±190.8)	4.11 (±0.18)
*A. pseudurbicum*	0.29 (±0.04)	1143.3 (±74.6)	na
*A. saundersii*	1.96 (±0.66)	503.3 (±23.0)	2.25 (±0.17)
*A. sedifolium*	3.93 (±0.72)	1385.1 (±132.5)	3.02 (±0.10)
*A. simsii*	0.51 (±0.08)	577.2 (±27.4)	1.85 (±0.10)
*A. stuessyi*	1.34 (±0.18)	1298.6 (±27.4)	2.71 (±0.13)
*A. tabuliforme*	1.35 (±0.39)	614.8 (±97.9)	3.52 (±0.21)
*A. undulatum*	0.66 (±0.47)	575.3 (±275.7)	1.18 (±0.06)
*A. urbicum*	1.40 (±0.25)	1025.9 (±36.4)	4.82 (±0.14)
*A. valverdense*	0.30 (±0.03)	1375.4 (±85.9)	2.99 (±0.16)
*A. volkeri*	0.18 (±0.07)	1163.9 (±119.6)	3.89 (±0.12)

na, not assessed.

### Correlation of nocturnal acid accumulation with minimum conductance and degree of succulence

**Fig. 3:**
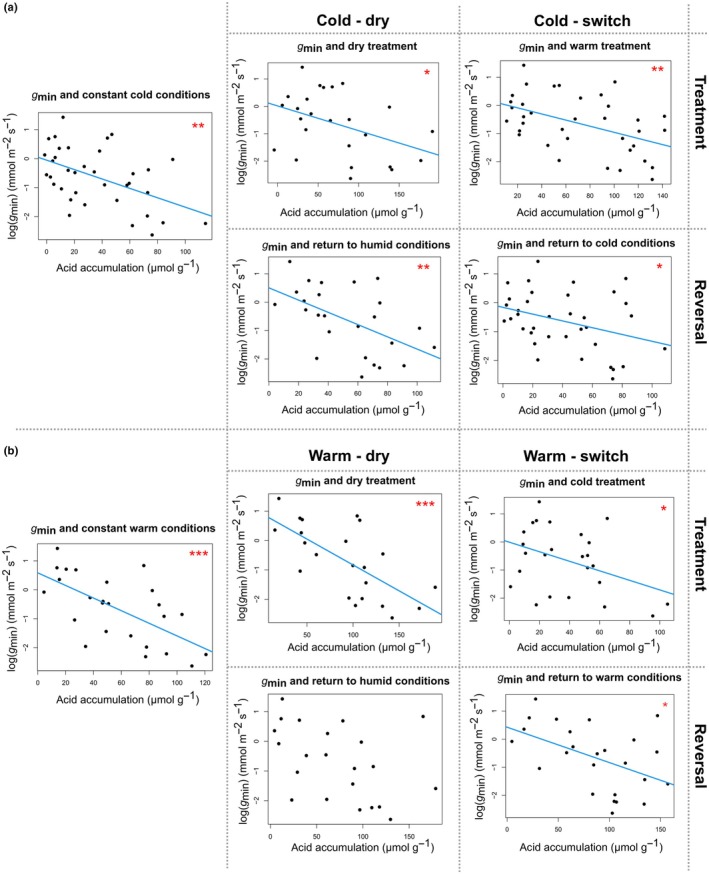
Scatter plots of natural logarithm of mean minimum conductance (*g*_min_) relative to mean nocturnal acid accumulation (ΔH^+^) in leaves of cold‐ (a) and warm‐acclimatised *Aeonium* plants (b) from the climate chamber experiment. Each data point represents one taxon. Constant cold or constant warm conditions in the left plot of each sub‐figure encompass all data of the respective control groups (all phases) plus acclimatisation phase of the respective drought and temperature treatment groups. Blue lines correspond to linear regressions for each scatter plot with significant correlation, and red asterisks indicate significance (*, 0.01 < *P* < 0.05; **, 0.001 < *P* < 0.01; ***, *P* < 0.001).

**Fig. 4:**
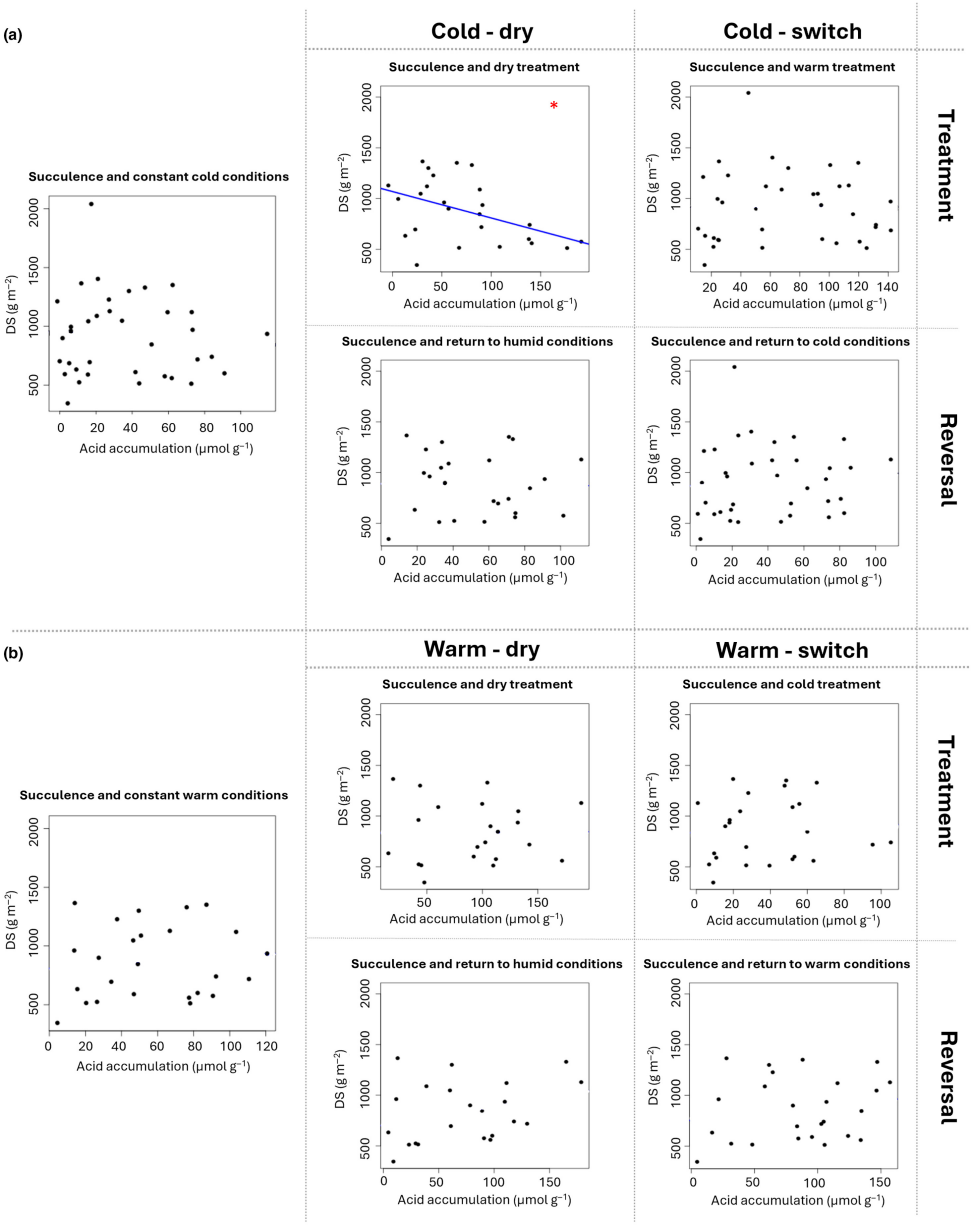
Scatter plots of mean degree of succulence (DS) relative to mean nocturnal acid accumulation (ΔH^+^) in leaves of cold‐ (a) and warm‐acclimatised *Aeonium* plants (b) from the climate chamber experiment. Each data point represents one taxon. Constant cold or constant warm conditions in the left plot of each sub‐figure encompass all data of the respective control groups (all phases) plus acclimatisation phase of the respective drought and temperature treatment groups. The blue line corresponds to the linear regression for the only significant correlation, and the red asterisk indicates this significance (*, 0.01 < *P* < 0.05).

Because the dataset of *g*_min_ was not normally distributed but instead approximately exponentially distributed, it was log‐transformed before linear regression analysis. Within all experimental phases except one (namely the reversal phase of warm – dry), mean ΔH^+^ was significantly negatively correlated with the logarithm of mean *g*_min_ (log (*g*_min_) in Fig. 3), which is in line with our expectation of lower *g*_min_ in species with stronger CAM activity. By contrast, mean DS and mean ΔH^+^ were not significantly correlated under any of the experimental conditions except the cold – dry treatment phase (Fig. 4). Surprisingly, this correlation was significantly negative (Fig. 4a), contradicting our hypothesis of a positive correlation of ΔH^+^ and DS. Here, the species with the highest ΔH^+^ (mean > 100 μmol g^−1^, that is, *A. arboreum* subsp. *holochrysum, A. balsamiferum*, *A. castello‐paivae*, *A. glandulosum*, and *A. gomerense*) all had relatively low values of DS (median < 750 g m^−2^). By contrast, CAM was not significantly induced by the drought treatment in the vast majority of those species with median DS above 750 g m^−2^ (Table 1; Fig. 2), meaning that these highly succulent plants were largely unaffected by the imposed water shortage. Because the maximum storage capacity of organic acids from nocturnal CO_2_ fixation is expected to be limited by succulence, we also searched for a correlation of mean DS and mean LT with maximum ΔH^+^ (instead of mean ΔH^+^), but did not find any significant correlation in either of these cases (Fig. S3).

### Trait evolution across the *Aeonium* phylogeny

In the following, the studied traits, that is, ΔH^+^, *g*_min_, and DS, are summarised clade by clade through the *Aeonium* phylogeny. Because LT was well correlated with DS (Fig. S2), it was not plotted in addition to DS. For ΔH^+^, we only compare those values measured at the end of the cold–switch treatment phase, that is, at the end of the heat treatment, because there is no missing data for this subset, and ΔH^+^ was most variable across taxa under these potentially CAM‐inducing conditions.

**Fig. 5:**
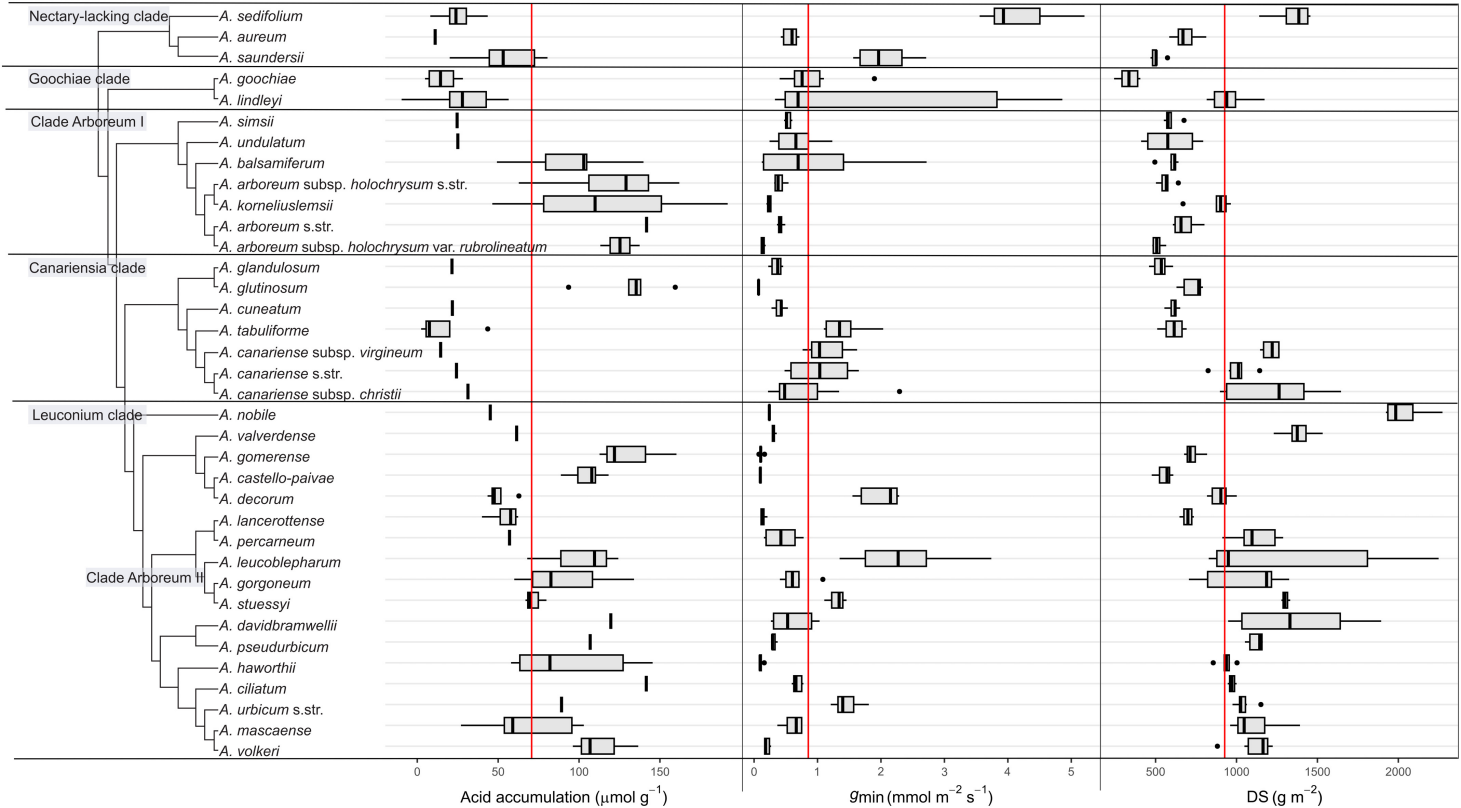
Phylogeny of *Aeonium* after Messerschmid *et al*. (2023) trimmed to contain only the taxa studied here. Three boxplots giving the measurement values for (from left to right) nocturnal acid accumulation (ΔH^+^) after the heat treatment, minimum conductance (*g*_min_), and degree of succulence (DS). Horizontal lines in each boxplot indicate the mean value of each trait taken across all taxa. Upper and lower whiskers indicate maximum and minimum observations within the 1.5‐fold of the interquartile range, respectively. Outliers beyond these ranges are given as dots.

The nectary‐lacking clade comprising *A. sedifolium* through *A. saundersii* was characterised by low ΔH^+^ (i.e. with mean consistently below average), but *g*_min_ and DS were heterogeneous between the three species (Fig. 5). The Goochiae clade of *A. goochiae* and *A. lindleyi* also featured low CAM activity (Fig. 5). The overall intermediate *g*_min_ values were much more variable in the significantly more succulent *A. lindleyi* compared to *A. goochiae* (Fig. 5). Clade Arboreum I comprises *A. simsii* and *A. undulatum* with below‐average CAM activity successively sister to a clade with some of the highest values of ΔH^+^ altogether, that is *A. balsamiferum* through *A. arboreum* subsp. *holochrysum* var. *rubrolineatum* (Fig. 5). The whole clade was characterised by below‐average *g*_min_ and DS. In the Canariensia clade, *A. glutinosum* from Madeira was the only species with above‐average ΔH^+^ while at the same time having the lowest value of *g*_min_ for any of the studied species, but only intermediate DS (Fig. 5). The three sampled subspecies of *A. canariense* had the highest DS in this clade and intermediate *g*_min_. The highest *g*_min_ in this clade, on the other hand, was found in *A. tabuliforme* with below‐average DS. In the Leuconium clade (*A. nobile* through *A. volkeri*) comprising sect. *Leuconium* and the three extra‐Canarian species of clade Arboreum II (*A. leucoblepharum* through *A. stuessyi*), relatively strong CAM expression, low *g*_min_, and a high DS were predominant. The most notable exception to this was *A. decorum* with below‐average ΔH^+^, intermediate DS, and above‐average *g*_min_. Other taxa deviated from this pattern, too, such as the highly succulent *A. nobile*, *A. percarneum*, and *A. valverdense* with surprisingly low levels of ΔH^+^, potentially due to the fact that only one plant was available for the heat treatment.

### Phylogenetic signal and phylogenetic generalised least squares (PGLS) regressions

Of all variables measured here, only DS, LT, and ΔH^+^ under one particular experimental condition (i.e. the cold – dry treatment) exhibited Pagel's λ that was significantly different from zero (Table 2). Significance was also found when considering the SE of these variables (not shown). These results suggest that the phylogenetic patterns of DS and LT, as measured for succulence, and of CAM induction by drought are better approximated by an evolutionary BM model than the other investigated variables. Mapping ΔH^+^ evolution onto the *Aeonium* phylogeny revealed overall greater values for Pagel's λ when plants were exposed to stressful experimental conditions (Fig. S4). Significance of λ to be different from zero was only found for the cold – dry treatment, while values for the warm – dry treatment had the second lowest *p*‐value (Table 2). For drought treatments, the effect of increased λ extended into the reversal phase (Fig. S4) during which plants again received water more regularly (see Fig. 1). Those clades with stronger CAM expression under the heat treatment, that is *A. balsamiferum* + *A. arboreum* s.l. and the Leuconium clade (treatment phase of group cold – switch in Fig. S4) also showcased increased CAM expression upon and beyond the drought treatment in the warm climate chamber (treatment and reversal phases of group warm – dry) and after being returned to high temperatures (reversal phase of group warm – switch). Conversely, those clades with weak or no CAM expression under the heat treatment, that is, the nectary‐lacking clade, Goochiae clade, *A. simsii* and *A. undulatum* of clade Arboreum I, and the Canariensia clade (see Fig. 5), also consistently had below‐average ΔH^+^ under the other stressful stages of the climate chamber experiment (Fig. S4).

**Table 2:** Phylogenetic signal (Pagel's λ) for minimum conductance (*g*_min_), degree of succulence (DS), leaf thickness (LT), and nocturnal acidification (ΔH^+^) under all phases of the climate chamber experiment.

Variable	Pagel's λ	LogL	*P*‐value
*g*_min_	0.44	−43.6	0.164
**DS**	**0.77**	**−259.2**	**0.023**
**LT**	**0.75**	**−47.1**	**0.001**
ΔH^+^ constant cold conditions	< 0.00	−174.1	1.000
**ΔH** ^**+**^ **cold – dry treatment**	**0.83**	**−130.8**	**0.027**
ΔH^+^ cold – dry reversal	< 0.00	−119.4	1.000
ΔH^+^ cold – switch treatment	< 0.00	−186.6	1.000
ΔH^+^ cold – switch reversal	< 0.00	−173.0	1.000
ΔH^+^ constant warm conditions	< 0.00	−124.3	1.000
ΔH^+^ warm – dry treatment	0.81	−115.3	0.116
ΔH^+^ warm – dry reversal	< 0.00	−117.0	1.000
ΔH^+^ warm – switch treatment	< 0.00	−115.6	1.000
ΔH^+^ warm – switch reversal	< 0.00	−129.3	1.000

Maximum likelihood values for λ significantly different from zero are shown in bold.

The PGLS regression analyses of ΔH^+^ under the heat treatment with all bioclimatic variables from the species occurrence data returned significant correlations for linear regressions with altogether five bioclimatic variables (BIO1, BIO5, and BIO8–BIO10), all of them temperature‐related, as well as with the potential solar radiation in summer (Fig. S5). Placement of the tree tips was strikingly similar in all five diagrams with these bioclimatic variables, that is annual mean temperature, maximum temperature of the warmest month, and mean temperatures of the wettest, driest and warmest quarter. In these cases, the correlation was significant and positive, meaning that higher ΔH^+^ was noted in species with natural distribution in hotter areas of the Canary Islands. The correlation with potential solar radiation in summer was significant and negative, conversely meaning that higher ΔH^+^ was noted in species with natural distribution in areas of the Canary Islands with lower potential solar radiation in summer. None of the PGLS regression analyses of *g*_min_ with bioclimatic variables were significant, but the strongest correlation (−0.187) of *g*_min_ was with BIO3, that is isothermality. This suggests that species with low *g*_min_ tend to occur in regions with relatively high day‐night temperature ranges relative to seasonal temperature ranges.

Finally, PGLS regression analyses of DS and ΔH^+^ as well as *g*_min_ and ΔH^+^ for each experimental condition separately revealed a significant negative effect for the warm – dry treatment phase in both cases (Table S4).

## Discussion

The integration of a wealth of traits is essential for plant fitness, and past studies have discovered examples for the correlation and correlated evolution of certain hydraulic traits of plants (Sanchez‐Martinez *et al*., 2020; Ávila‐Lovera *et al*., 2023). As a water‐saving metabolic pathway, CAM is expected to be functionally integrated in plant–water relations and was shown to be correlated with specific other hydraulic and anatomical traits (Males, 2016; Niechayev *et al*., 2019; Leverett *et al*., 2023b). The functional integration of traits likely changes with the expression of the individual traits and is furthermore constrained by ancestral trait expression. We here demonstrated experimentally the CAM activity of 32 *Aeonium* species, which covers 78% of the species diversity of the genus and reveals a broad range of CAM expression from nearly pure C_3_ photosynthesis (*A. aureum* and *A. canariense* subsp. *virgineum*) to weak facultative CAM (e.g. *A. canariense* s.str., *A. goochiae*, *A. sedifolium* and *A. tabuliforme*), strong facultative CAM (e.g. *A*. *arboreum*, *A. decorum* and *A. volkeri*), and constitutive CAM (e.g. *A. balsamiferum*, *A. castello‐paivae* and *A. glutinosum*). At the same time, we measured *g*_min_ which ranged from 0.07 ± 0.00 mmol m^−2^ s^−1^ (*A. glutinosum*) to 3.93 ± 0.72 mmol m^−2^ s^−1^ (*A. sedifolium*) across the same set of species, and DS which ranged from 349.7 ± 96.6 g m^−2^ (*A. goochiae*) to 1983.8 ± 154.6 g m^−2^ (*A. nobile*, Table 1).

### Minimum conductance (*g*_min_) is a novel CAM‐related trait

A new finding filling a knowledge gap in the context of the correlated evolution of CAM‐related traits is a significant negative correlation of CAM expression and log(*g*_min_) for nearly all our experimental treatments (Fig. 3). Our results indicate a strong functional interdependence of low *g*_min_ and high CAM expression that we characterise in the following. When CAM activity is induced, facultative CAM plants almost invariably express a reduction of stomatal conductance during mid‐day (see gas exchange patterns of facultative CAM plants in Winter & Holtum (2014) and Winter (2019)). This reduction of stomatal conductance to water vapour increases the proportion of cuticular conductance to overall conductance (Burghardt & Riederer, 2003). Therefore, the role of the cuticle as a transpiration barrier becomes more important with decreasing stomatal conductance during the day, which is typical for CAM. Cuticular conductance (and thereby potentially *g*_min_) is largely dependent on the chemical composition of the cuticular waxes (e.g. Leide *et al*., 2007), which in turn is dictated by a set of wax biosynthesis genes (Yeats & Rose, 2013). Our hypothesis of lower *g*_min_ with increasing reliance on nocturnal CO_2_ assimilation by means of CAM was underpinned for *Aeonium* under almost all conditions imposed in our climate chamber experiment, including stressful conditions like the warm – dry treatment phase but also constant cold and well‐watered conditions (Fig. 3).

Although there was an apparent scarcity of low log(*g*_min_) data points in the range of low ΔH^+^ (lower left corner of diagrams in Fig. 3), this does not mean that weak CAM or even C_3_ photosynthesis is less compatible with a highly effective transpiration barrier. Indeed, there are many C_3_ plants with highly effective cuticular transpiration barrier properties (e.g. Helbsing *et al*., 2000 (*Anthurium salvinii*, Araceae); Riederer, 2006 (*Ficus elastica*, Moraceae); Jetter & Riederer, 2016 (*Monstera deliciosa*, Araceae)). However, half of the species with the lowest values of leaf minimum conductance (*g*_min_ < 1.3 × 10^−6^ m s^−1^, corresponding to *c*. 0.053 mmol m^−2^ s^−1^) measured up until the most recent review of *g*_min_ and cuticular conductance that included succulent plants (Schuster *et al*., 2017) belong to genera with well‐documented CAM activity (Gilman *et al*., 2023). Surprisingly few CAM or succulent plant species have been tested for leaf or stem *g*_min_. Of the 160 plant species that had been tested for leaf *g*_min_ between 1996 and 2017, only 13 belonged to genera with CAM, all of them being tropical epiphytes or vines, with the exception of *Clusia flava* and *Zamioculcas zamiifolia* (Schuster *et al*., 2017). All of them ranged within the lowest third of all observations of *g*_min_. In addition, *Aeonium glutinosum*, the species with the lowest *g*_min_ in our study, ranks number 15 of the top‐most effective transpiration barriers, and in an unpublished study of succulent members of tribe Senecioneae (Asteraceae) by the authors, three species (i.e. *Senecio medley‐woodii*, *S. melastomifolius*, and *S. meuselii*) were identified that are only surpassed in transpiration barrier effectiveness by *Zamioculcas zamiifolia* (Karbulková *et al*., 2008). All this strongly suggests that succulent plants are yet understudied in terms of cuticular properties and could represent the life form group with the most effective transpiration barrier properties. This is supported by the fact that many epiphytes belonging to the life form group with the lowest values of *g*_min_ ever observed (Schuster *et al*., 2017) are indeed succulent plants.

### Succulence is a fundamental CAM‐related trait with contrasting relationships to CAM expression under drought and heat

In contrast to *g*_min_, succulence has been studied in relation to CAM activity by many authors (e.g. de Santo *et al*., 1983; Silvera *et al*., 2005; Herrera, 2020). Succulence has been generally defined as the presence of an internal water storage that allows plants ‘to be temporarily independent from external water supply’ (Eggli & Nyffeler, 2009), and consequently, there are multiple ways to measure and quantify succulence. In addition to LT, we here take DS (Delf, 1912) as a reliable and easy‐to‐measure quantity which is known to be of limited use for plants with storage succulence (Herrera, 2020) and was rarely used in investigations of CAM and succulence, probably for this reason. However, this potential pitfall of leaf‐area‐based succulence plays hardly any role in *Aeonium* because their leaves are consistently of an all‐cell succulence type (Jimenez *et al*., 1983; Liu, 1989; Melo‐de‐Pinna *et al*., 2016; TM, pers. obs.).

We furthermore tried repeating the analyses with a dry‐weight ‐based measure for succulence (SWC_meas_, saturated water content, that is, the quotient of leaf saturated water mass and leaf dry mass; Ogburn & Edwards, 2012) that accounts for the species' physiological potential to take up water. However, we dismissed it mostly because it did not significantly correlate with LT (Fig. S2), possibly due to short rehydration periods (overnight, see Materials & methods), and SWC_meas_ did not reflect the apparent succulence of certain *Aeonium* species. For example, the evidently most succulent representative of the genus, *A. nobile*, with up to centimetre‐thick leaves, only ranked number 11 among the highest values of SWC_meas_, being surpassed by clearly less succulent species such as *A. tabuliforme*.

A surprising result here is that under constant cold or constant warm conditions, DS is not correlated with ΔH^+^ in *Aeonium* (Fig. 4). We found no significant positive correlation of ΔH^+^ and DS. Instead, we found a significantly negative relationship of ΔH^+^ and DS for the cold – dry treatment phase (Fig. 4a). However surprising this finding may be, contradicting our initial hypothesis, there is a possibility that the highly succulent species, which featured only intermediate nocturnal acidification in this treatment, did not experience the imposed drought as severely as the less succulent species, of which many strongly increased their CAM activity (Fig. 2). Another possible explanation for this negative correlation could be a dilution effect from a slightly hydrenchymatous tissue in the core of more succulent leaves that would increase in impact with increasing succulence (Leverett *et al*., 2023c). Interestingly, a similar, differential CAM response to drought in species with higher or lower levels of succulence was not paralleled when heat and drought were combined (Fig. 4b). Here, again, the relationship of ΔH^+^ and succulence was non‐significant.

While a positive relationship between CAM and indices of succulence has been shown by numerous earlier studies (Barrera Zambrano *et al*., 2014; Herrera, 2020; Leverett *et al*., 2023c; Gilman *et al*., 2024), our result here suggests a less general association. Succulence has been proposed to enable CAM activity once it has reached a certain threshold value (e.g. Edwards, 2023). Our results suggest that this threshold should be low relative to the succulence spectrum in *Aeonium* and thus met by most *Aeonium* species, because some of the highest ΔH^+^ values were measured in only moderately succulent leaves (Fig. 4). Values of DS far beyond this putative threshold also do not seem to significantly enhance the potential, maximum CAM activity in *Aeonium* (Fig. S3). Herrera (2020), in a meta‐analysis of leaf anatomical traits and leaf carbon isotope ratios (δ^13^C), found a nonlinear positive correlation of δ^13^C with leaf thickness, indicating the possibility of variable leaf thickness in accordance with high δ^13^C (i.e. constitutive or strong CAM) but a preclusion of very thick leaves with low δ^13^C (i.e. weak or facultative CAM). This is well in line with the often‐proposed selection pressure against C_3_ photosynthesis in strong succulents due to massively reduced mesophyll conductance in succulent plants and thereby reduced availability of CO_2_ for carbon fixation by ribulose‐1,5‐bisphosphate carboxylase/oxygenase (Rubisco; Maxwell *et al*., 1997; Earles *et al*., 2018; Edwards, 2019; Leverett *et al*., 2023a). We here do not investigate δ^13^C (as in Herrera, 2020) which would be indicative of the lifetime CAM activity of photosynthetic organs (O'Leary, 1988), but instead quantify CAM activity by ΔH^+^, a short‐term indicator of nocturnal primary carbon fixation for plants with CAM. This may explain why we indeed find a substantial proportion of relatively low values of ΔH^+^ in species with highly succulent leaves, at least under unstressed conditions (Fig. 4). Interestingly, the scarcity of C_3_‐like δ^13^C values in combination with very thick leaves is less pronounced in Crassulaceae compared to tropical epiphytes (see fig. 3 in Herrera, 2020). This may indicate an unknown coping mechanism with limitations to daytime carbon fixation imposed by succulence in Crassulaceae, a family that may showcase the oldest instance of CAM evolution in any extant plant lineage (Sage *et al*., 2023).

### The capacity for CAM performance under drought is phylogenetically conserved

The variation of ΔH^+^ for the cold – switch treatment phase throughout the *Aeonium* phylogeny (Fig. 5) clearly shows that the nectary‐lacking clade and Goochiae clade, successive sister to the rest of *Aeonium*, have a lower capacity for nocturnal acid accumulation or inducibility of CAM. A higher capacity for CAM performance evolved in one branch of clade Arboreum I, in *A. glutinosum*, and in the Leuconium clade (Fig. 5). Among earlier studies of CAM evolution in *Aeonium*, Pilon‐Smits *et al*. (1992) concluded a very similar scenario of increasing CAM activity in more derived lineages because these authors combined a largely accurate phylogenetic hypothesis with measurements of enzyme activity on top of the more common δ^13^C measurements. By mapping ΔH^+^ onto the *Aeonium* phylogeny for all treatments of our cultivation experiment separately, we here show that the phylogenetic signal (Pagel's λ) was strongest for the drought treatments (Fig. S4) but significantly different from zero only under the cold – dry treatment (Table 2). This indicates that the capacity for CAM expression under drought stress may evolve in a more or less random manner covarying with shared branch lengths of related species, while CAM expression under all other experimental conditions was more or less independent from phylogenetic relationships (λ very close to zero; Table 2). In line with this, the phylogenetic position of obligate CAM species (i.e. those species with higher ΔH^+^ even under well‐watered conditions) shows little phylogenetic signal in *Aeonium* (see cold – switch and cold – dry acclimatisation phase in Fig. S4). This may indicate a higher lability of the obligate CAM condition in *Aeonium*. This observation might be explained by the large diversity of ecological niches on the Canary Islands which *Aeonium* has filled rapidly during the past 8–4 Myr (dos Santos *et al*., 2022; Messerschmid *et al*., 2023) and by possible hybridisation events of lineages with contrasting CAM performances. We hypothesise that selection against obligate CAM might occur in situations where, at least for a part of the growing season, C_3_ photosynthesis is more productive. In that way, competition with fast‐growing C_3_ plants would be possible during the generally wetter winter months (which is the main growing season of *Aeonium*) while growth could be continued in a CAM mode throughout the drier summer period, although several *Aeonium* species largely suspend their growth in summer by forming closed buds from their rosettes (*Aeonium* sect. *Greenovia*) or by reducing their rosettes to a compressed core (e.g. in *A. arboreum* s.l.).

Dos Santos *et al*. (2022) showed that *Aeonium* does not colonise the driest habitats of the Canary Islands, although the genus occupies 86% of the available climatic niche space on the islands. In fact, in those lowland regions that are inhabitable for *Aeonium* due to pronounced aridity, hardly any vascular plants can be found (dos Santos *et al*., 2022). Furthermore, despite being a classic example of insular adaptive radiation (Lems, 1960; Jorgensen & Frydenberg, 1999) where one would expect a high rate of niche adaptation across species, *Aeonium* was recently shown to exhibit a surprisingly moderate rate of niche evolution (dos Santos *et al*., 2024) on the basis of a large occurrence dataset and complete species‐level phylogeny. The most pronounced cases of morphological diversification were found for within‐island speciation events, possibly representing sympatric speciation (Messerschmid *et al*., 2023), but in many instances, slight changes in reproductive traits or phenology were most probably the reason for reproductive isolation and speciation (dos Santos *et al*., 2024). CAM photosynthesis as a flexible and plastic physiological trait (Dodd *et al*., 2002) has likely been essential for the vast ecological niche diversification in the evolution of *Aeonium*, as already proposed by Lösch (1990), and is shown here to respond in distribution to several temperature‐related bioclimatic variables as well as solar radiation in summer (Fig. S5). This is paralleled by our observation that the baseline CAM activity of *Aeonium* plants acclimatised to their current environmental conditions is a phylogenetically unstable trait (λ for ΔH^+^ close to zero in Table 2) that has likely undergone a high rate of diversification. At the same time, the capacity for CAM performance under drought stress is more conserved throughout the *Aeonium* phylogeny (Table 2) and is therefore probably more constrained by the prevailing ancestral conditions of succulence and *g*_min_. We here conclude that *g*_min_ was adapted in concert with CAM physiology to meet the environmental demands of the different climatic niches that *Aeonium* has managed to occupy.

## Competing interests

None declared.

## Author contributions

TFEM and GK conceived the study. TFEM and SEH performed the experiments. TFEM, JMV, SEH, and JAB analysed the data. TFEM and GK wrote the initial draft of the manuscript. All authors interpreted the results and contributed to manuscript writing.

## Disclaimer

The New Phytologist Foundation remains neutral with regard to jurisdictional claims in maps and in any institutional affiliations.

## Supporting information


**Fig S1** Photographs of various *Aeonium* leaves to demonstrate leaf morphological diversity.
**Fig. S2** Linear regression analyses of degree of succulence (DS; g m^−2^) and saturated water content (SWC; g g^−1^) against leaf thickness (LT; mm).
**Fig. S3** Linear regression analyses of mean degree of succulence (DS; g m^−2^), mean leaf thickness (LT; mm) and mean minimum conductance (gmin; mmol m^−2^ s^−1^) against maximum nocturnal acid accumulation (ΔH^+^_max_).
**Fig. S4** Continuous trait mapping and phylogenetic signal (Pagel's λ) of nocturnal acidification (ΔH^+^) onto the *Aeonium* phylogeny for all experimental phases and groups except controls.
**Fig. S5** Phylogenetic generalised least squares (PGLS) regression analyses of nocturnal acidification (ΔH^+^) after the heat treatment (i.e. treatment phase of group cold – switch) with bioclimatic variables and seasonal potential solar radiation from species occurrence data.
**Table S1** Accessions of plants used in the climate chamber experiment.

**Table S2** Titratable acidity (μmol g^−1^) of all samples from the climate chamber experiment and corresponding ΔH^+^ values.

**Table S3** Minimum conductance (*g*_min_; m s^−1^), degree of succulence (DS; g m^−2^), saturated water content (SWC; g g^−1^) and leaf thickness (LT; mm) of all sampled leaves.

**Table S4** Results of the phylogenetic generalised least squares (PGLS) regression analyses of minimum conductance (gmin; m s^−1^) and degree of succulence (DS; g m^−2^) against nocturnal acidification (ΔH^+^; μmol g^−1^) of leaves for each condition of the climate chamber experiment.
Please note: Wiley is not responsible for the content or functionality of any Supporting Information supplied by the authors. Any queries (other than missing material) should be directed to the *New Phytologist* Central Office.

## Data Availability

All raw data and R scripts are deposited on Github (https://github.com/thibaud‐ipen/Aeonium‐CAM‐gmin).
