# Supplementary material for: Stronger expression of crassulacean acid metabolism (CAM) requires effective cuticular transpiration barriers but not necessarily strong succulence
Source: New Phytol. 2026 Jan 23;249(6):2760–75. doi: 10.1111/nph.70909 (PMC12917466; doi:10.1111/nph.70909)
Supplement: Supplementary file 1 — Fig S1 Photographs of various Aeonium leaves to demonstrate leaf morphological diversity. Fig. S2 Linear regression analyses of degree of succulence (DS; g m−2) and saturated water content (SWC; g g−1) against leaf thickness (LT; mm). Fig. S3 Linear regression analyses of mean degree of succulence (DS; g m−2), mean leaf thickness (LT; mm) and mean minimum conductance (gmin; mmol m−2 s−1) against maximum nocturnal acid accumulation (ΔH+ max). Fig. S4 Continuous trait mapping and phylogenetic signal (Pagel's λ) of nocturnal acidification (ΔH+) onto the Aeonium phylogeny for all experimental phases and groups except controls. Fig. S5 Phylogenetic generalised least squares (PGLS) regression analyses of nocturnal acidification (ΔH+) after the heat treatment (i.e. treatment phase of group cold – switch) with bioclimatic variables and seasonal potential solar radiation from species occurrence data. Table S1 Accessions of plants used in the climate chamber experiment. [file NPH-249-2760-s003.docx]

## *New Phytologist* Supporting Information

Article title: Stronger expression of Crassulacean Acid Metabolism (CAM) requires effective cuticular transpiration barriers but not necessarily strong succulence

Authors: Thibaud F. E. Messerschmid, Jurriaan M. de Vos, Susanne E. Hamburger, Jessica A. Berasategui, Gudrun Kadereit

Article acceptance date: 02 December 2025

**
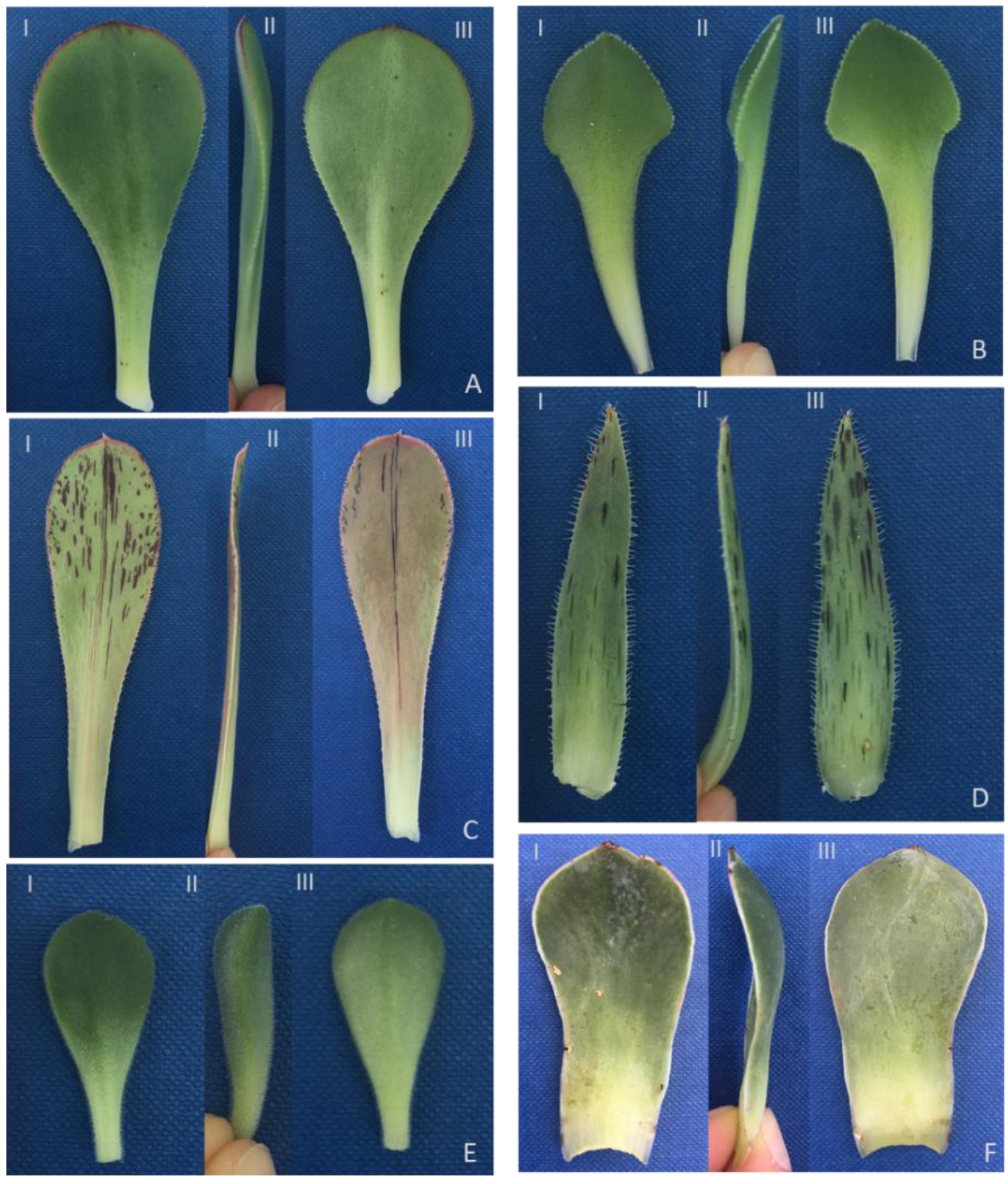
**

**Fig. S1** Photographs of various *Aeonium* leaves to demonstrate leaf morphological diversity. Those taxa that were used to infer leaf shrinkage curves are represented here: (**a**) *Aeonium volkeri*, (**b**) *A. glandulosum*, (**c**) *A. arboreum* subsp. *holochrysum* var. *rubrolineatum*, (**d**), *A. simsii*, (**e**) *A. lindleyi* subsp. *lindleyi*, (**f**) *A. aureum*. Each leaf was photographed from the following sides: (**I**) adaxial, (**II**) lateral and (**III**) abaxial.


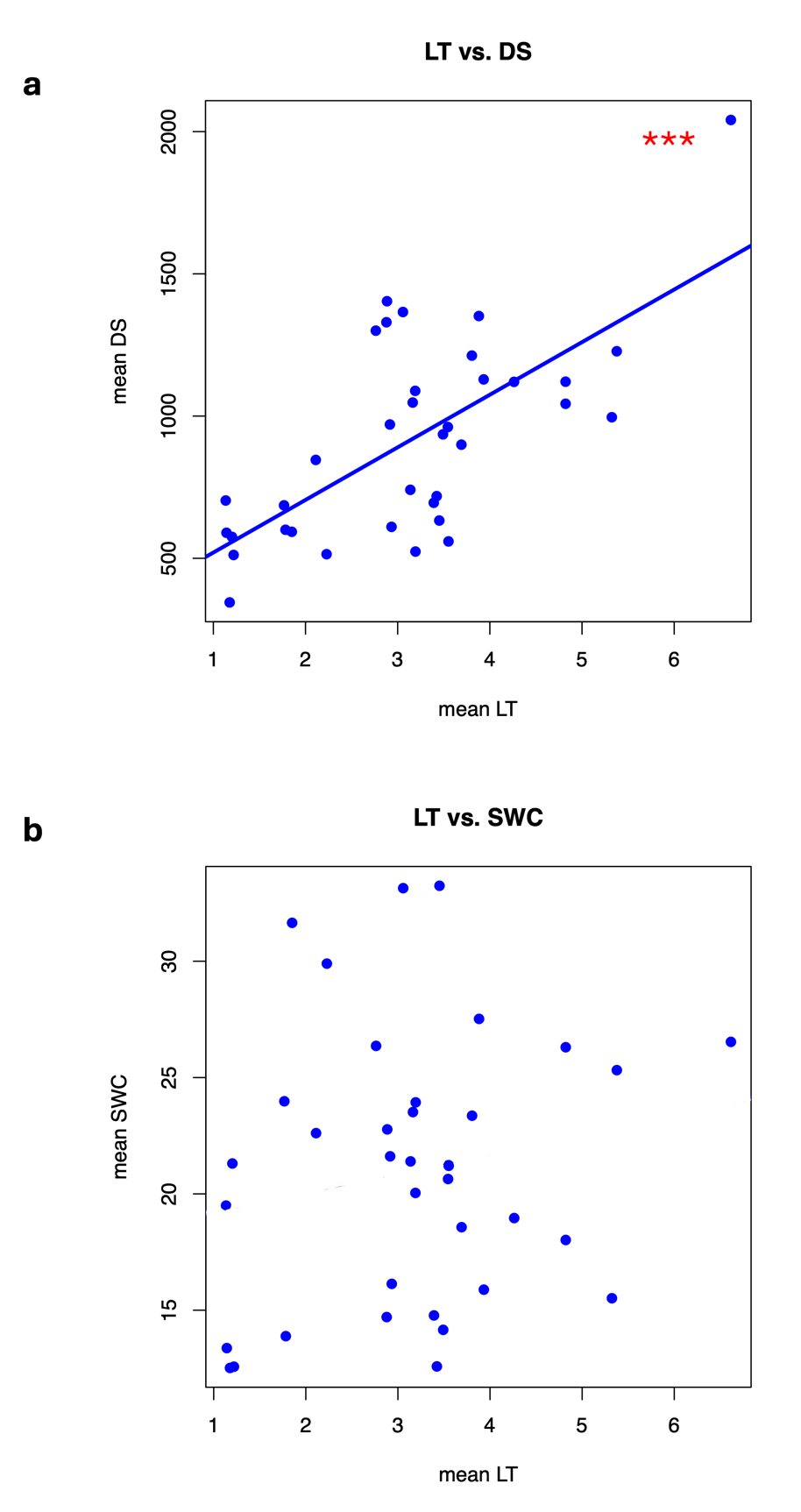


**Fig. S2** Linear regression analyses of (**a**) degree of succulence (DS; g m^-2^) and (**b**) saturated water content (SWC; g g^-1^) against leaf thickness (LT; mm). Each data point represents paired mean values for one taxon. Correlation was significant for (**a**) *p* < 0.001, but non-significant for (**b**) *p* = 0.288.


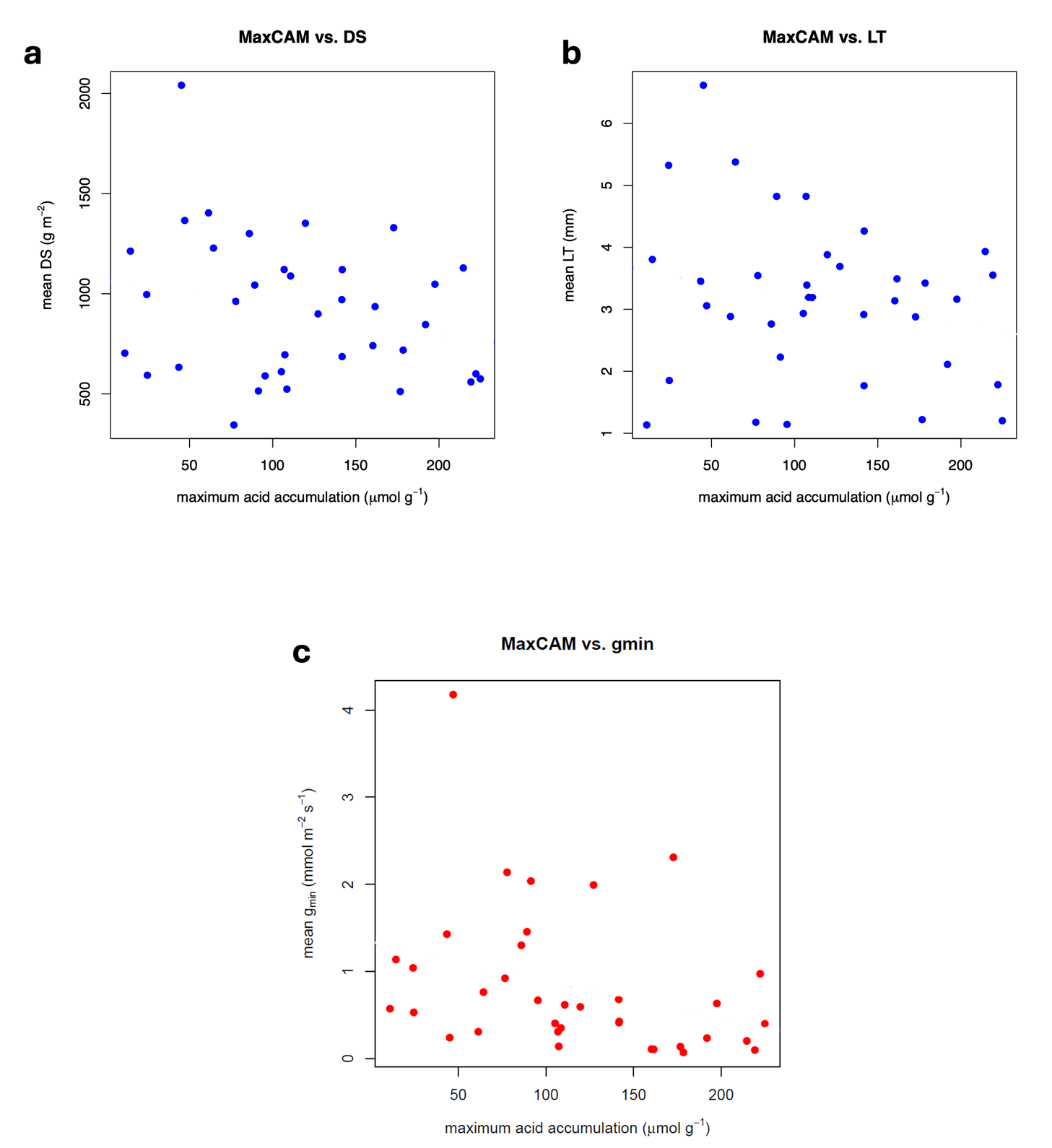


**Fig. S3** Linear regression analyses of (**a**) mean degree of succulence (DS; g m^-2^), (**b**) mean leaf thickness (LT; mm) and (**c**) mean minimum conductance (*g*_min_; mmol m^-2^ s^-1^) against maximum nocturnal acid accumulation (ΔH^+^_max_). Each data point represents one taxon. Correlation was non-significant for all analyses: (**a**) *p* = 0.149, (**b**) *p* = 0.185, (**c**) *p* = 0.055.


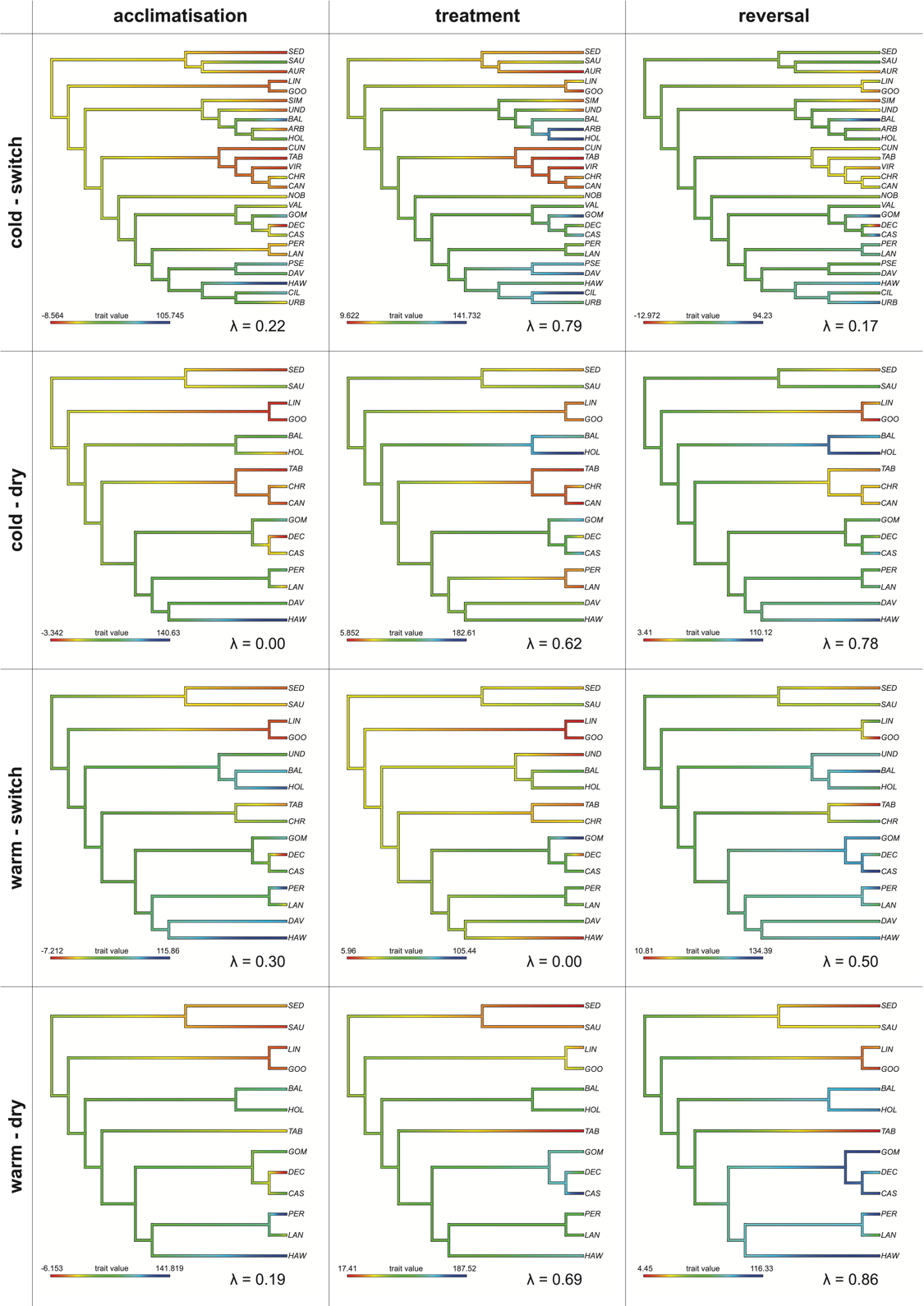


**Fig. S4** Continuous trait mapping and phylogenetic signal (Pagel’s λ) of nocturnal acidification (ΔH^+^) onto the *Aeonium* phylogeny for all experimental phases and groups except controls. The phylogenies are pruned to only contain taxa that underwent the respective treatments and were included in the occurrence data set. Taxon names are abbreviated by the first three letters of each epithet of lowest taxonomic hierarchy. The heat of the colour spectrum indicates CAM expression with red signifying low ΔH^+^ and blue signifying high ΔH^+^.


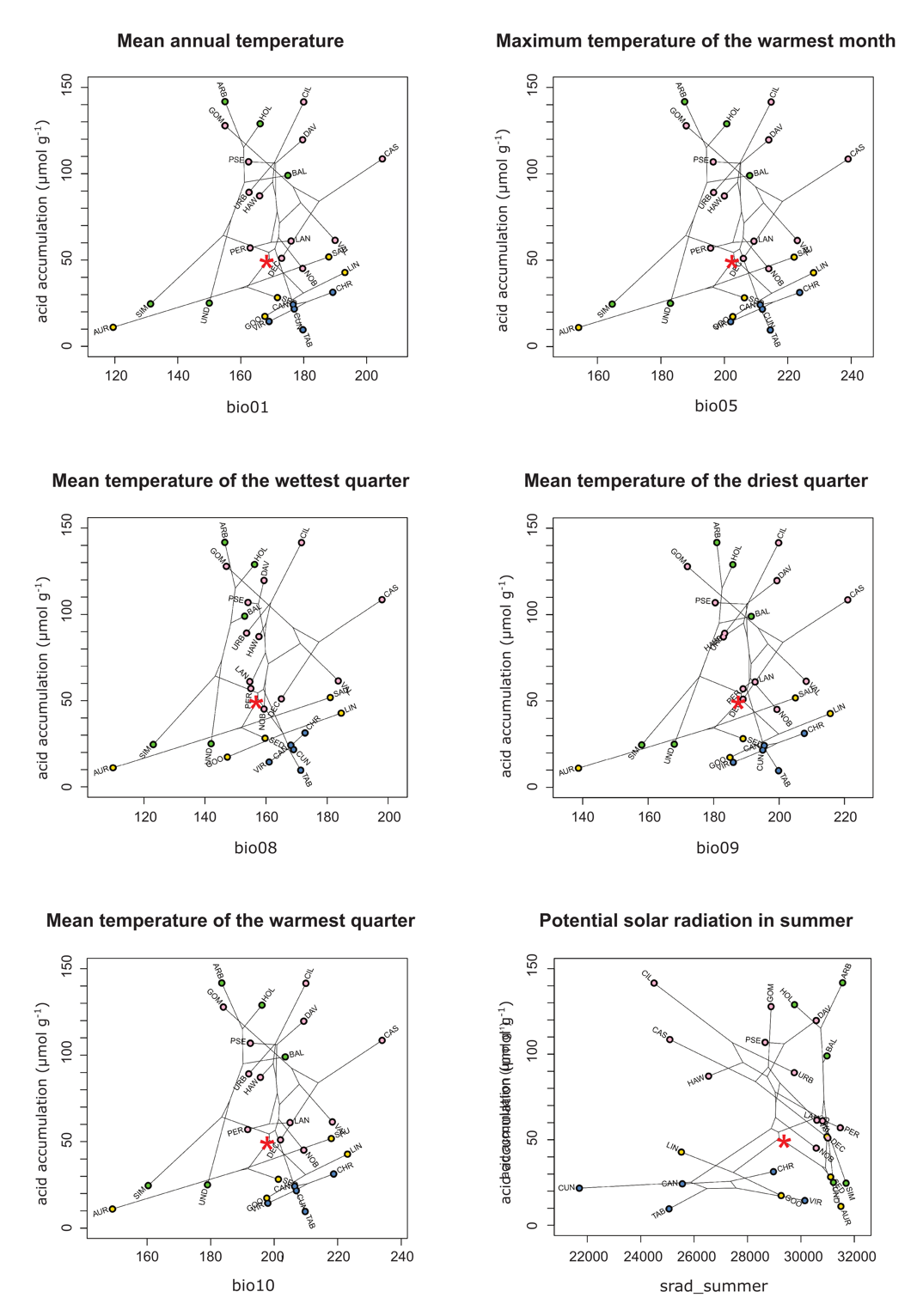


**Fig. S5** Phylogenetic generalised least squares (PGLS) regression analyses of nocturnal acidification (ΔH^+^) after the heat treatment (i.e., treatment phase of group cold - switch) with bioclimatic variables and seasonal potential solar radiation from species occurrence data. Only significant regressions are shown. Taxon names are abbreviated by the first three letters of each epithet of lowest taxonomic hierarchy. Tip colours indicate clade affiliation for each taxon (yellow: nectary-lacking clade and Goochiae clade; green: clade Arboreum I; blue: Canariensia clade; purple: Leucosedum clade).

**Table S1** Accessions of plants used in the climate chamber experiment.

| Species | Section | Accession  number^*^ | Origin | Number of replicates | Growth form^+^ |
| --- | --- | --- | --- | --- | --- |
| *Aeonium arboreum* Webb & Berthel. subsp. *arboreum* | *Aeonium* | 2021/1719 | Gran Canaria, leg. Lösch | 1 | shrub or subshrub |
| *A. arboreum* subsp. *holochrysum* (H.Y.Liu) Bañares var. *holochrysum* | *Aeonium* | 2021/1772 | BG of the University of Mainz | 29 | shrub or subshrub |
| *A. arboreum* subsp. *holochrysum* var. *rubrolineatum* (Svent.) H.Y.Liu | *Aeonium* | not further cultivated | BG of the University of Mainz | 8 | shrub or subshrub |
| *A. aureum* (C.Sm. ex Hornem.) T.Mes | *Greenovia* | 1109 (BG Bonn) | BG of the University of Bonn | 1 | branching rosette plant |
| *A. balsamiferum* Webb & Berthel. | *Aeonium* | 2021/1795 | BG of the University of Mainz, leg. Lösch | 29 | shrub or subshrub |
| *A. canariense* (L.) Webb & Berthel. subsp. *canariense* | *Canariensia* | not further cultivated | Tenerife, near Las Bodegas, leg. Messerschmid et al. | 2 | branching rosette plant |
| *A. canariense* subsp. *christii* (Burchard) Bañares | *Canariensia* | not further cultivated | La Palma, leg. dos Santos & Brilhante | 4 | branching rosette plant |
| *A. canariense* subsp. *virgineum* (Webb) Bañares | *Canariensia* | 2021/1783 | Gran Canaria, between Teror & San Mateo, leg. W. Rauh | 1 | branching rosette plant |
| *A. castello-paivae* Bolle | *Leuconium* | not further cultivated | La Gomera, leg. Schmitz | 30 | shrub or subshrub |
| *A. ciliatum* Webb & Berthel. | *Leuconium* | 2021/1730 | Tenerife, near Las Bodegas, leg. Messerschmid et al. | 1 | shrub or subshrub |
| *A. cuneatum* Webb & Berthel. | *Canariensia* | 048167924 (BGBM Berlin) | Tenerife, Sierra de Anaga | 2 | branching rosette plant |
| *A. davidbramwellii* H.Y.Liu | *Leuconium* | 2021/1733 | La Palma, Volcan Teneguia, leg. H. Grasmück | 5 | shrub or subshrub |
| *A. decorum* Webb ex Bolle | *Leuconium* | 142591 (BG Heidelberg) | La Gomera, San Sebastián, leg. W. Rauh | 30 | shrub or subshrub |
| *A. glandulosum* (Aiton) Webb & Berthel. | *Patinaria* | 1014/3169 | BG Munich-Nymphenburg | 8 | monocarpic rosette plant (Liu, 1989) |
| *A. glutinosum* (Aiton) Webb & Berthel. | *Pittonium* | 2021/1742 | Madeira, leg. Lösch | 30 | shrub or subshrub (Liu, 1989) |
| *A. gomerense* (Praeger) Praeger | *Leuconium* | not further cultivated | BG of the University of Mainz | 18 | shrub or subshrub |
| *A. goochiae* Webb & Berthel. | *Goochiae* | 2021/1745 | BG of the University of Mainz | 16 | shrub or subshrub |
| *A. gorgoneum* J.A.Schmidt | *Aeonium* | 2021/1748 | Santo Antão, leg. Lösch | 13 | shrub or subshrub (Liu, 1989) |
| *A. haworthii* Webb & Berthel. | *Leuconium* | 2021/1750 | Tenerife, leg. Lösch | 24 | shrub or subshrub |
| *A. korneliuslemsii* H.Y.Liu | *Aeonium* | 2021/1717 | Morocco, Djbel Imzi, leg. Erpenbach & Levejohann | 17 | shrub or subshrub (Liu, 1989) |
| *A. lancerottense* (Praeger) Praeger | *Leuconium* | 2021/1754 | Lanzarote, Famara massif, leg. E. Royl | 21 | shrub or subshrub |
| *A. leucoblepharum* Webb ex A.Rich. | *Aeonium* | 2021/1756 | Yemen, Sumara pass, leg. Lavranos & Newton | 14 | shrub or subshrub (Liu, 1989) |
| *A. lindleyi* Webb & Berthel. subsp. *lindleyi* | *Goochiae* | 2021/1761 | Tenerife, coordinates available, leg. J. Los | 18 | shrub or subshrub |
| *A. lindleyi* subsp. *viscatum* (Bolle) Bañares | *Goochiae* | 2021/1760 | La Gomera, Valle San Sebastián, leg. W. Rauh | 34 | shrub or subshrub |
| *A. mascaense* Bramwell | *Leuconium* | 2021/1762 | Tenerife, leg. Lösch | 28 | shrub or subshrub (Bramwell, 1982) |
| *A. nobile* (Praeger) Praeger | *Leuconium* | not further cultivated | La Palma, leg. dos Santos & Brilhante | 1 | monocarpic rosette plant |
| *A. percarneum* (Murray) Pit. & Proust | *Leuconium* | 2021/1764 | Gran Canaria, between Carrizal de Tejeda and El Toscón, leg. dos Santos & Brilhante | 6 | shrub or subshrub |
| *A. pseudurbicum* Bañares | *Leuconium* | not further cultivated | Tenerife, Masca, leg. Messerschmid | 1 | monocarpic rosette plant |
| *A. saundersii* Bolle | *Petrothamnium* | 2021/1767 | BG of the University of Bonn | 33 | shrub or subshrub |
| *A. sedifolium* (Webb ex Bolle) Pit. & Proust | *Petrothamnium* | 2021/1768 | Tenerife, Guía de Isora, leg. W. Rauh | 23 | shrub or subshrub |
| *A. simsii* (Sweet) Stearn | *Aeonium* | 81 2745 /1 (ZSS Zurich) | Sukkulenten-Sammlung Zurich | 1 | branching rosette plant |
| *A. stuessyi* H.Y.Liu | *Aeonium* | 2021/1776 | Ethiopia, Lalibela, leg. E. Fischer | 14 | shrub or subshrub (Liu, 1989) |
| *A. tabuliforme* Webb & Berthel. | *Canariensia* | not further cultivated | Tenerife, leg. Lösch | 29 | monocarpic rosette plant |
| *A. undulatum* Webb & Berthel. | *Aeonium* | 140772 (BG Heidelberg) | Gran Canaria, Tejeda, leg. W. Rauh | 3 | shrub or subshrub |
| *A. urbicum* (C.Sm. ex Hornem.) Webb & Berthel. | *Leuconium* | 201010201 (BG Mainz) | BG of the University of Mainz | 1 | monocarpic rosette plant |
| *A. valverdense* (Praeger) Praeger | *Leuconium* | 012018420 (BGBM Berlin) | Berlin Botanic Garden and Botanical Museum | 1 | shrub or subshrub |
| *A. volkeri* E.Hern. & Bañares | *Leuconium* | 2021/1784 | Tenerife, Chamorga, leg. H. Grasmück | 18 | shrub or subshrub (Hernández & Bañares, 1996) |

* cultivated at the Botanic Garden Munich-Nymphenburg if not stated otherwise

+ following dos Santos et al. (2022) if not stated otherwise
